# Clinically‐relevant proteomic and metabolomic signatures with effective neuroprotective treatment of the Tg4344AD rat model of Alzheimer's disease

**DOI:** 10.1002/alz70856_103515

**Published:** 2025-12-26

**Authors:** Kalyani Chaubey, Lijun Dou, Edwin Vázquez‐Rosa, Coral Cintrón‐Pérez, Kathryn Franke, Jaymie Voorhees, Feixiong Cheng, Andrew A. Pieper

**Affiliations:** ^1^ Institute of Transformative Molecular Medicine, Case western Reserve University, Cleveland, OH, USA; ^2^ Brain Health Medicines Center, Harrington Discovery Institute, University Hospitals Cleveland Medical Center, Cleveland, OH, USA; ^3^ Case Western Reserve University, Cleveland, OH, USA; ^4^ Louis Stokes Cleveland VA Medical Center, Cleveland, OH, USA; ^5^ Cleveland Clinic, Cleveland, OH, USA; ^6^ Inotiv, St. Louis, MO, USA; ^7^ Cleveland Clinic Genome Center, Cleveland, OH, USA

## Abstract

**Background:**

Alzheimer's disease (AD) is a progressive brain disorder that severely impairs behavior and cognitive functions. This includes depression, which often appears earlier than cognitive impairment. This study reports early‐stage proteomic and metabolomic signatures associated with AD in the TgF344AD rat model of AD, at a time when rats display depression but not cognitive impairment. The results are compared to human AD proteomic and metabolomic databases to determine clinical relevance of the findings.

**Method:**

Nine‐month‐old TgF344AD rats, at an early stage of AD when they display depression‐like behavior but are not yet cognitively impaired, were treated daily for six months with either vehicle or the neuroprotective compound P7C3‐S243. This compound has been shown to stabilize NAD^+^ homeostasis in models of AD, and this course of treatment was previously reported to prevent the development of neuropsychiatric symptoms in this rat model. We analyzed brain proteomics using label‐free proteomics via UPLC‐MS/MS on a Q‐Exactive Orbitrap HF‐X system and conducted metabolomics analysis through Metabolon. The obtained signatures were then compared to human AD proteomic and metabolomic databases to identify similar directional changes, and major molecular mechanisms were elucidated.

**Results:**

We identified a total of 904 proteins that significantly changed at the proteomic level and 95 metabolites that were significantly altered at the metabolomic level, when comparing TgF344AD rats to their wild type littermates. Many of these proteins and metabolites exhibited changes in the same direction in human AD brain proteomic and metabolomic databases. Furthermore, many of the AD‐associated signatures that changed in both humans and rats were normalized towards wild‐type levels after six months of daily treatment with P7C3‐S243 administered to TgF344AD rats.

**Conclusion:**

We have identified common and unique molecular signatures of AD at the proteomic and metabolomic levels that are held in common across the TgF344 AD rat model and the human AD brain. We additionally determined which of the aberrant expression patterns held in common across rat and human AD brain are normalized by a neuroprotective treatment that prevents the onset of disease in these rats. This points us towards new insight into mechanisms underlying effective prevention of AD.

